# Current and Potential Future Seasonal Trends of Indoor Dwelling Temperature and Likely Health Risks in Rural Southern Africa

**DOI:** 10.3390/ijerph15050952

**Published:** 2018-05-10

**Authors:** Thandi Kapwata, Michael T. Gebreslasie, Angela Mathee, Caradee Yael Wright

**Affiliations:** 1 Environment and Health Research Unit, South African Medical Research Council, Johannesburg 2028, South Africa; Thandi.kapwata@mrc.ac.za (T.K.); angela.mathee@mrc.ac.za (A.M.); 2 Department of Geography, Geoinformatics and Meteorology, University of Pretoria, Pretoria 0028, South Africa; 3 School of Agriculture, Earth, and Environmental Sciences, University of KwaZulu-Natal, Durban 3629, South Africa; Gebreslasie@ukzn.ac.za; 4 Department of Environmental Health, Faculty of Health Sciences, University of Johannesburg, Johannesburg 2028, South Africa; 5 Environment and Health Research Unit, South African Medical Research Council, Pretoria 0084, South Africa

**Keywords:** climate change, rural setting, current and predicted future health risks, environmental health, South Africa

## Abstract

Climate change has resulted in rising temperature trends which have been associated with changes in temperature extremes globally. Attendees of Conference of the Parties (COP) 21 agreed to strive to limit the rise in global average temperatures to below 2 °C compared to industrial conditions, the target being 1.5 °C. However, current research suggests that the African region will be subjected to more intense heat extremes over a shorter time period, with projections predicting increases of 4–6 °C for the period 2071–2100, in annual average maximum temperatures for southern Africa. Increased temperatures may exacerbate existing chronic ill health conditions such as cardiovascular disease, respiratory disease, cerebrovascular disease, and diabetes-related conditions. Exposure to extreme temperatures has also been associated with mortality. This study aimed to consider the relationship between temperatures in indoor and outdoor environments in a rural residential setting in a current climate and warmer predicted future climate. Temperature and humidity measurements were collected hourly in 406 homes in summer and spring and at two-hour intervals in 98 homes in winter. Ambient temperature, humidity and windspeed were obtained from the nearest weather station. Regression models were used to identify predictors of indoor apparent temperature (AT) and to estimate future indoor AT using projected ambient temperatures. Ambient temperatures will increase by a mean of 4.6 °C for the period 2088–2099. Warming in winter was projected to be greater than warming in summer and spring. The number of days during which indoor AT will be categorized as potentially harmful will increase in the future. Understanding current and future heat-related health effects is key in developing an effective surveillance system. The observations of this study can be used to inform the development and implementation of policies and practices around heat and health especially in rural areas of South Africa.

## 1. Introduction

There has been a growing interest in the relationship between temperature in indoor and outdoor settings, especially in the context of climate change and projected increases in extreme temperature events [1]. The exposure of individuals to temperature extremes is affected not only by outdoor temperature, but also by temperature experienced in the indoor environment. Indoor conditions represent an important microenvironment because of the amount of time people spend at home; they provide a suitable representation of temperature exposure experienced by dwelling occupants [2]. The elderly, children, and disabled people are particularly vulnerable to extreme indoor temperatures and may experience heat stress or heatstroke, among other heat-related health outcomes [3,4]. Measures of exposure to heat vary and include maximum, minimum, average temperatures, or apparent temperature (AT), where the latter refers to a ‘real-feel’ metric that includes the influence of relative humidity with temperature [5,6,7].

Climate change has resulted in rising temperature trends which have been associated with changes in temperature extremes globally [8,9,10,11]. World leaders who attended the 21st Conference of the Parties (COP 21) agreed to strive to limit the rise in global average temperatures to below 2 °C compared to industrial era conditions, and to aim for a target of 1.5 °C [12]. However, on average, global average temperatures are projected to exceed these temperature goals, with increases of between 1.4 °C and 5.8 °C expected by the end of the year 2100 [13]. This phenomenon is expected to exacerbate the negative health impacts of heat caused by higher temperatures and increased frequency and severity of heatwaves [14].

Current research suggests that Africa is experiencing longer, hotter, and more frequent heatwaves that are occurring over larger areas, compared to Europe and other continents, for the same time periods [15]. Studies further show that in the future, the African region will be subjected to more intense heat extremes in a shorter time horizon, with projections predicting 20–80 more heat-wave days occurring annually over subtropical southern Africa and increases of 4–6 °C in annual average maximum temperatures for southern Africa during 2088–2099 [16,17,18].

Negative health effects of exposure to raised temperatures during heatwaves and on hot days include cramps, fainting, heat exhaustion, heatstroke, and dehydration [19,20,21]. Increased temperatures may exacerbate existing chronic ill health conditions such as cardiovascular disease, respiratory disease, cerebrovascular disease, and diabetes-related conditions [22,23]. Exposure to extreme temperatures has also been associated with mortality [24,25].

Evidence suggests that people of lower socio-economic status are at an increased risk of adverse heat-related effects [26]. Populations in economically disadvantaged settings are more vulnerable because they lack the resources to cope and adapt to heatwaves [27]. There has been little published research on exposure to elevated temperatures and health impacts in developing countries; existing studies particularly in South Africa are largely confined to occupational settings [28,29,30,31,32,33,34]. Knowledge on the temperature environments in which people live (and work) and possible health impacts associated with high temperature is required to guide development and design of prevention and awareness programs and policy recommendations with the purpose of reducing heat-associated morbidity and mortality. This is of particular importance in rural residential settings where poor communities do not have access to heat warnings and recommended coping mechanisms, access to health care services is usually limited and health facility resources are inadequate [35].

Therefore, this study aimed to consider the relationship between indoor and outdoor environments in rural residential settings in a current climate and warmer predicted future climate. Specific study objectives were to (a) measure and then assess the impact of outdoor temperatures on the indoor residential temperature microclimate in a rural setting in Limpopo province, South Africa; (b) explore seasonal trends in the indoor residential temperature microclimate; and (c) to consider possible health risks associated with indoor temperatures using AT as a measure of exposure. We took this approach to determine both current (measured) and future (predicted) possible indoor and outdoor temperature profiles in rural homes using the representative concentration pathway (RCP) 8.5 scenario of the fifth assessment report (AR5) of the Intergovernmental Panel on Climate Change (IPCC) [36,37]. This scenario predicts increases in the region of 2.6–4.8 °C by the end of the 21st century [38]. In a subsample of the sampled houses, we also assessed the influence of type of roof on indoor temperatures in both current and future temperature scenarios to suggest recommendations for coping mechanisms in relation to rural roofs and a warming climate.

## 2. Materials and Methods

### 2.1. Study Area

The study area was Limpopo province, South Africa. Limpopo has 74.4% of its households located in rural areas compared to the national average of 27.1% [39]. The province also has the highest level of poverty in the country with 78.9% of the total population of the province living below the national poverty line of ZAR 441 per person per month [40]. ZAR is the international code for the South African currency, the Rand. Studies have identified Limpopo province as being particularly vulnerable to climate change impacts due to its exposure to extreme weather events that include heat extremes [41,42,43,44].

This study was conducted in 406 households in the Greater Giyani local municipality located in Limpopo province. The municipality has a population size of 247,657 consisting of 57,537 households, the majority of which are located in several rural villages [39]. Within this municipality, 406 dwellings were randomly selected from four villages to participate in the study. A cluster sampling method was used. Ethics clearance for the study was granted by the South African Medical Research Council Research Ethics Committee (clearance no. EC005-3/2014, 9 May 2017) and permission was sought from the provincial, local and traditional leadership in the study area.

### 2.2. Outdoor Weather Data

Hourly measurements for daily ambient temperature, humidity and wind speed data were obtained from the South African Weather Service (SAWS) for the monitoring station located in Giyani, Limpopo province. Data were provided from 1 September 2016 to 30 September 2017 in an Excel format. SAWS has procedures in place for data quality assurance and quality control. These include removing impossible values resulting from human error or instrument malfunction, outliers and duplicated values [45].

### 2.3. Indoor Temperature and Relative Humidity Data

Temperatures and relative humidity inside the 406 houses were monitored and recorded using LogTag temperature loggers. Three separate sampling campaigns were conducted, one for each season (spring, summer, winter). For the spring and summer campaigns, one logger was placed in a room in each of the selected 406 dwellings. Temperature and relative humidity were recorded at one-hour intervals during spring (5 September 2016 to 30 November 2016) and summer (1 December 2016 to 28 February 2017). The same variables were recorded at 2-h intervals during the winter campaign (7 July 2017 to 31 August 2017) in a sub-sample of 98. Initially, a total of 105 houses were randomly selected however, 7 (6.7%) did participate in the study. The logger was placed in a room frequently used by household occupants and affixed to a wall or high piece of furniture with double-sided tape to minimize tampering and to be as unobtrusive as possible in the home. Temperature was measured in degrees Celsius and relative humidity was measured as a percentage out of 100, data were downloaded from loggers into Microsoft Excel (2016) format. Before analysis, data were checked for inconsistencies and other anomalies and outliers (unrealistic values) were removed, where after data from all households were merged into a single file for further analysis. Temperature measurements were recorded correctly by the logtags. However, 0.13% (1280 out of 99,784 hourly data points) of the relative humidity data was omitted due to values being unrealistic.

### 2.4. Apparent Temperature

AT is an adjustment to the ambient temperature based on the level of relative humidity and it is deemed a measure of how humans actually perceive or feel temperature. AT is the most frequently used indicator of probable human physical reaction to weather conditions and several studies have used AT to examine the association between health and high temperature [46,47,48,49]. Therefore, hourly AT for each house was calculated using the equations below [50]

AT = Ta + 0.33 × e − 0.70 × ws − 4.00(1)

where

Ta = dry bulb temperature (°C)
e = water vapour pressure (hPa)
ws = wind speed (m/s) at an elevation of 10 m (set to 0 because this was an indoor setting)

e = rh/100 × 6.105 × exp (17.27 × Ta/(237.7 + Ta))(2)

where rh = relative humidity (%).

Currently, no comprehensive relationships between AT or temperature ranges and their effect on human health are available for Africa [46]. Therefore, an international symptom table developed by the United States National Weather Service (USNWS) and the National Oceanic and Atmospheric Administration (NOAA) was used in this study. This table has been used in similar studies on the African context [46]. Calculated indoor ATs were compared to USNWS NOAA heat health effects as shown in Table 1 [51] to estimate potential temperature ranges to which household occupants may be exposed and consider the temperature-associated health risks.

### 2.5. Household Questionnaire

Following written, informed consent, a pre-structured questionnaire was administered to a respondent from each of the 406 households participating in the summer indoor temperature measurement campaign. A household member of at least 18 years of age provided information on socio-economic and demographic status, specifically number of people in the household (explained as the people who eat a meal together), average monthly household income, number of people receiving various grants, perceived level of comfort in the house during hot weather and use of a fan and/or air conditioner during hot weather. During the wintertime indoor temperature monitoring campaign, a household member from the sub-sample of 98 households was asked about the roof type of their home.

### 2.6. Data and Statistical Analyses

All statistical analyses were performed in STATA version 14 (StataCorp, College Station, TX, USA) [52]. Data recorded by LogTags were linked to household questionnaire data by a unique household identifier number. AT was calculated for indoor and outdoor environments using Equations (1) and (2) above to evaluate possible health risks. Multiple linear regression was used to assess the association between indoor AT (dependent variable) and ambient outdoor temperature and AT (independent variables) to identify the most important predictor of indoor AT. Thereafter, to determine possible future risks resulting from indoor AT, linear regression was used to predict indoor AT based on predicted outdoor temperature. Predicted outdoor temperature values were obtained by using the projected increases in monthly temperature for South Africa from the Representative Concentration Pathways (RCP 8.5) climate model for the period 2080–2099 [36,37,53,54]. The projected data obtained from the RCP climate model is presented at a 1° × 1° global grid spacing, produced through bi-linear interpolation [55]. Statistical tests were also performed to assess statistical differences in temperatures according to roof type and between indoor and outdoor AT. A 5% level of significance was applied to all regression analysis.

## 3. Results

### 3.1. Household Characteristics

A large percentage of sampled households were below the upper-bound poverty line (i.e., ZAR 1077) as defined by Statistics South Africa in the year 2015 [54] (Table 2). Many households supplemented their income with state-sponsored social grants that support vulnerable groups. These grants were for children under the age of 18 (282 children) and elderly people over the age of 60 years (142 elderly). Most households appear to be single family homes with 87.9% of houses having between one and seven occupants.

**Figure 1:**
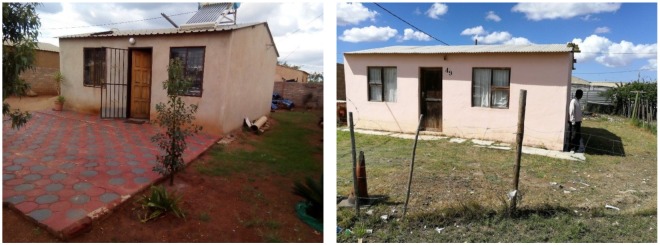
Houses in study site of Giyani, Limpopo province, South Africa (Source: Google Images from Gumtree.co.za).

Most of the sampled households were formal structures (92.6%) therefore further analysis by housing type was not conducted even though this is likely an important variable that should be considered in future research. Although not asked in the questionnaire, most houses were single story units built with brick and mortar with areas ranging from 36 m^2^ to 45 m^2^ (Figure 1).

**Figure 2:**
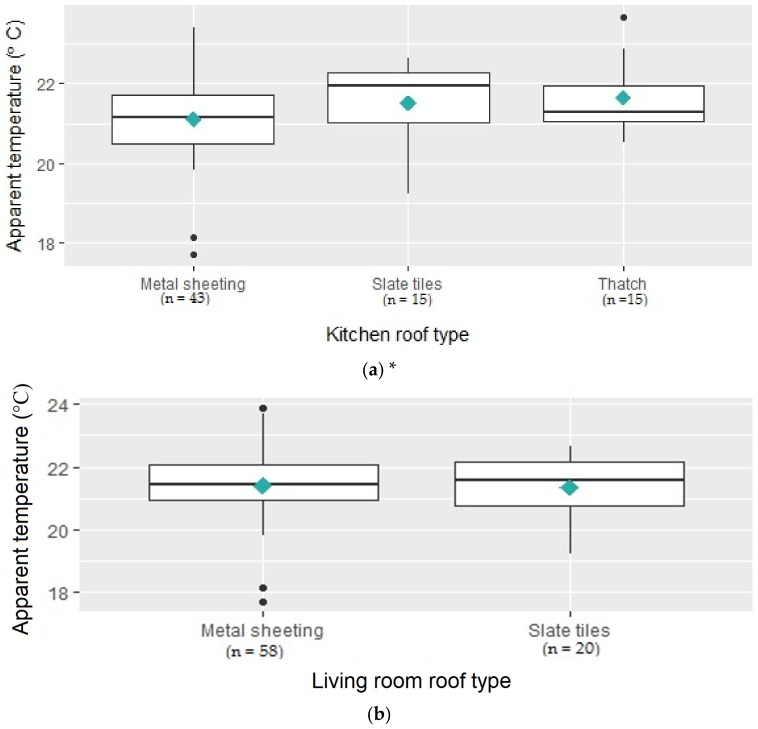
Mean hourly indoor AT calculated for 98 homes by roofing type from 7 July 2017 to 31 August 2017 (line: median; interquartile range box: middle 50% of the data; whiskers extending from either end of the box: ranges for the bottom and top 25% of the data values; solid black dots: outliers; diamond dot: mean), * Responses captured as other were excluded from plot (*n* = 8), N across plots does not add up to 98 due to missing responses. (**a**) Shows results for kitchen roof type and (**b**) for living room roof type.

In the winter subsample of 98 households, where roofing type was captured, the most common roofing type in both the kitchens and the living rooms was metal sheets (43.9% and 59.2% of homes, respectively). Figure 2a shows that mean hourly indoor AT during winter was slightly higher in kitchens with thatched roofs (21.7 °C) compared to other roof types. A one-way ANOVA analysis showed that there was a statistically significant difference between the three roof types (*p* < 0.001). A Tukey post hoc test revealed that temperatures were statistically significantly higher in kitchens with thatched roofs compared to kitchens with metal sheets (*p* < 0.001). However, the difference between temperatures in kitchens with thatched roofs and kitchens with slate tiles was not statistically significant (*p* > 0.10). AT was also higher in living rooms with metal sheet roofing (21.4 °C) compared to slate tiles (Figure 2b). A *t*-test revealed that that difference was not statistically significant (*p* > 0.10).

### 3.2. Current Seasonal Trends in Indoor and Outdoor Temperatures

The seasons are defined according to recommendations by the South African Weather Service [56]. Spring is from 1 September to 30 November; summer is from 1 December to 28/29 February; and winter is from 1 June to 31 August. Table 3 provides a summary of ambient and indoor meteorological conditions recorded during summer, spring, and winter. Mean AT was highest in summer for both outdoor and indoor environments, 33.8 °C and 32.2 °C, respectively. The highest maximum outdoor temperature and highest maximum calculated AT both occurred in summer (41.3 °C and 52.8 °C, respectively). Heat-related illnesses, and in extreme events deaths, have been associated with ambient temperatures above 35 °C [57,58]. Outdoor temperatures exceeded 35 °C for 43 days during the study period (236 days), with most of these days occurring in summer and spring.

**Figure 3:**
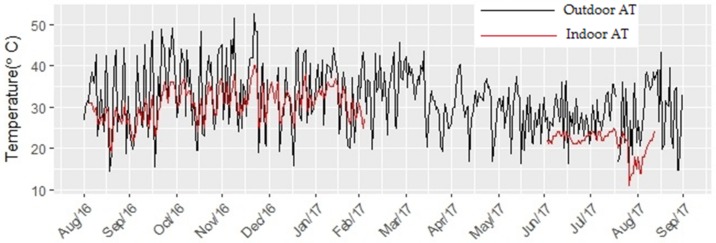
Daily hourly outdoor and indoor apparent temperature calculated during spring, summer, and winter in Limpopo Province, South Africa.

Outdoor AT exceeded indoor AT by about 3.6 °C for the duration of the study period (Figure 3). Daily average outdoor AT and indoor AT displayed pronounced seasonal fluctuations; both were higher in summer months (December to February) and spring (September to November) compared to winter (July to August). A two-sample *t*-test showed that the difference between indoor and outdoor AT was statistically significant (*p* < 0.001).

### 3.3. Current Likely Health Risks Based on AT Calculations of Indoor Temperatures

**Figure 4:**
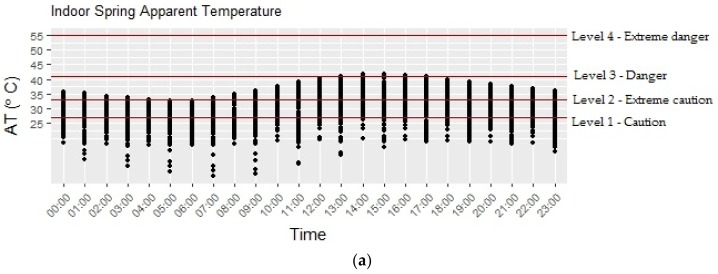

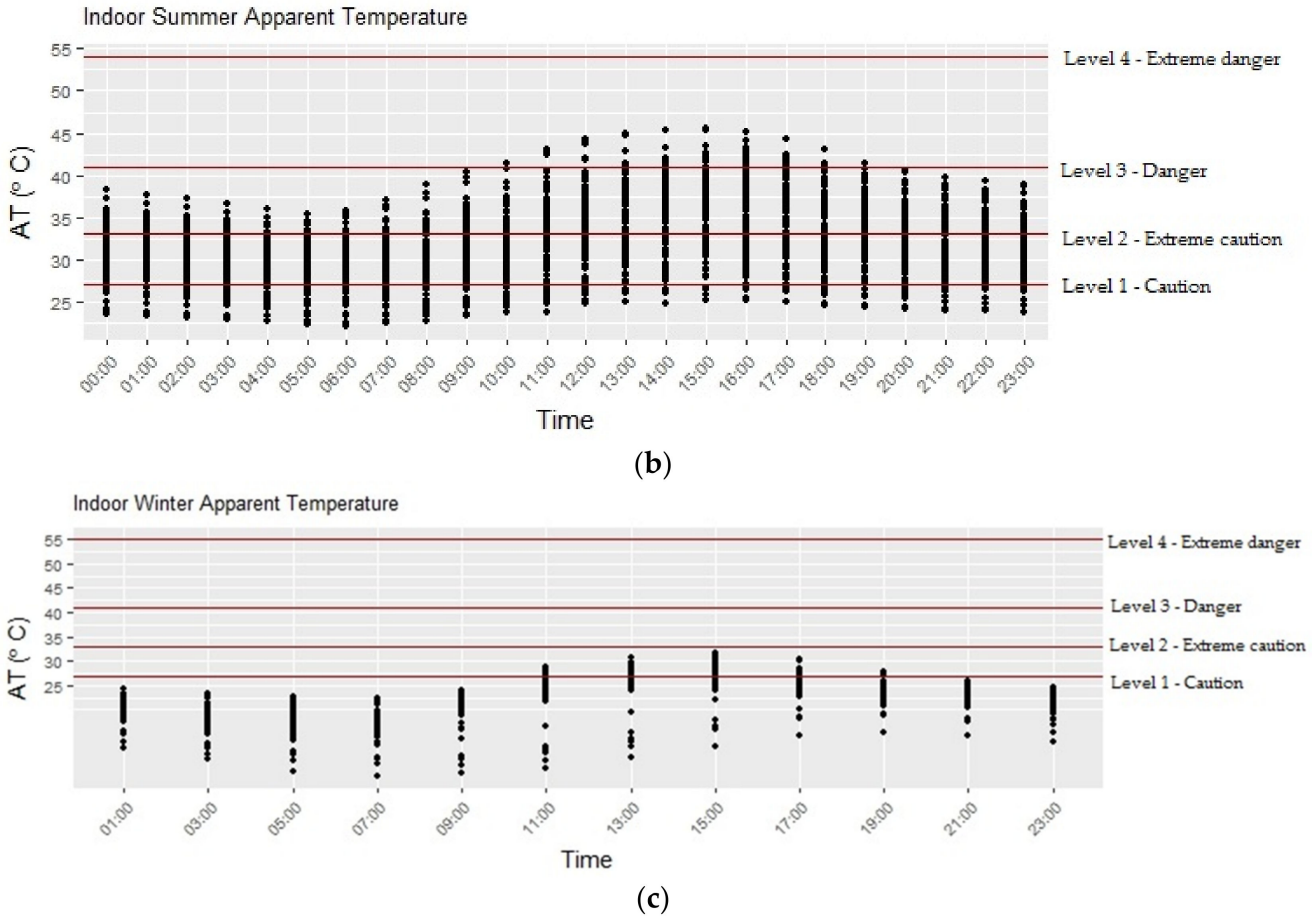
Average hourly indoor apparent temperature with superimposed symptom bands as described in Table 1 for spring (**a**), summer (**b**) and winter (**c**). Dots represent individual households.

Daily indoor AT was within each of the symptom bands in Table 1 for several days during the study period; 78 days were within the caution band and 61 days were within the more severe extreme caution band. Figure 4a–c illustrate the diurnal patterns of indoor AT calculated for summer, spring, and winter. Indoor AT gradually increased throughout the day, peaking between 1:00 p.m. and 3:00 p.m. on most days. It then began to decrease after 4:00 p.m. AT in most homes generally appeared to fall within the higher symptom bands (caution and extreme caution) from mid-morning to early afternoon during all seasons. During most hours of the day in summer, indoor AT was classified as caution or extreme caution. AT was also classified as being in the danger symptom band between 11:00 a.m. and 6:00 p.m. in several homes during the summer season. During spring, fewer hours than in summer were within the more severe symptom bands; however, some homes had a few hours within the extreme caution band. In winter, the highest symptom band to which AT rose was the caution symptom band between 11:00 a.m. and 5:00 p.m.

### 3.4. Potential Future Temperature and Associated Heat-Health Risks

To identify if ambient temperature and AT can be used to predict indoor conditions that dwelling occupants could experience, multiple linear regression was used to analyze the association between indoor AT and ambient temperature and AT. The multiple regression model explained 45.2% of the variance in indoor AT (*p* < 0.001). Results presented in Table 4 show that outdoor temperature was most statistically significant in explaining indoor AT (β = 0.55, *p* = 0.001).

Regression analysis was conducted for each season and for all seasons combined. The model that explained the highest percentage of variance in indoor apparent temperature and was statistically significant was the one with the data for all seasons combined therefore this is the model is reported.

**Figure 5:**
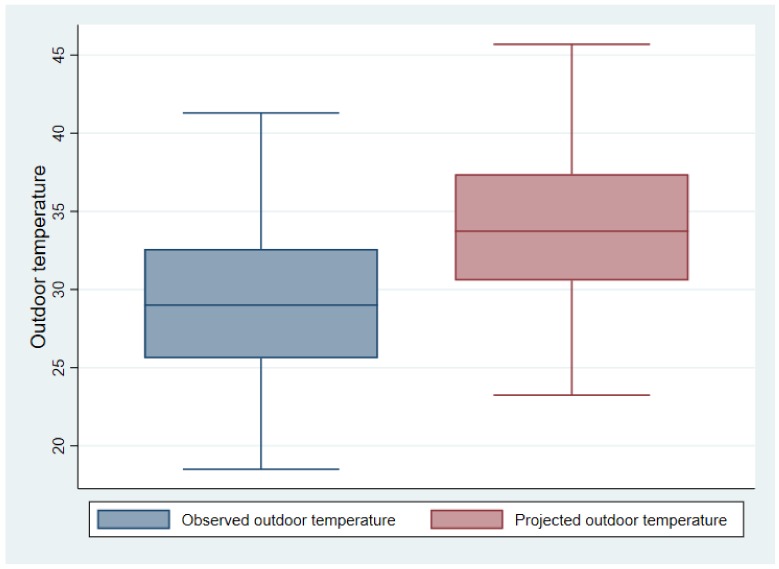
Observed outdoor temperature during the study period compared to outdoor temperatures projected using RCP 8.5 climate model.

RCP 8.5 projected increases in monthly temperature were added to the outdoor temperature recorded during the study period to determine possible future estimates of ambient temperature for the study site. Based on RCP projections for South Africa, average ambient temperature in the study area in Limpopo province was estimated to increase by a mean of 4.6 °C (Figure 5). This is consistent with findings by Engelbrecht et al. (2015) that annual average temperatures in South Africa will increase by 4–6 °C over the time period 2071–2100.

**Figure 6:**
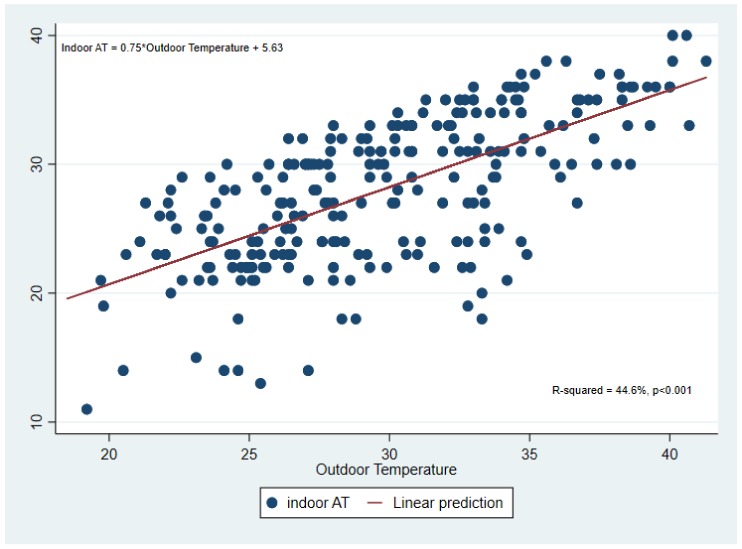
Linear regression between calculated indoor AT (using measured indoor temperature) and measured outdoor temperature for the period September 2016 to August 2017.

Based on the findings from the multiple regression that outdoor temperature is a significant predictor of indoor AT, linear regression was used to develop a prediction model for estimating future indoor AT from projected outdoor temperature (Figure 6).

**Figure 7:**
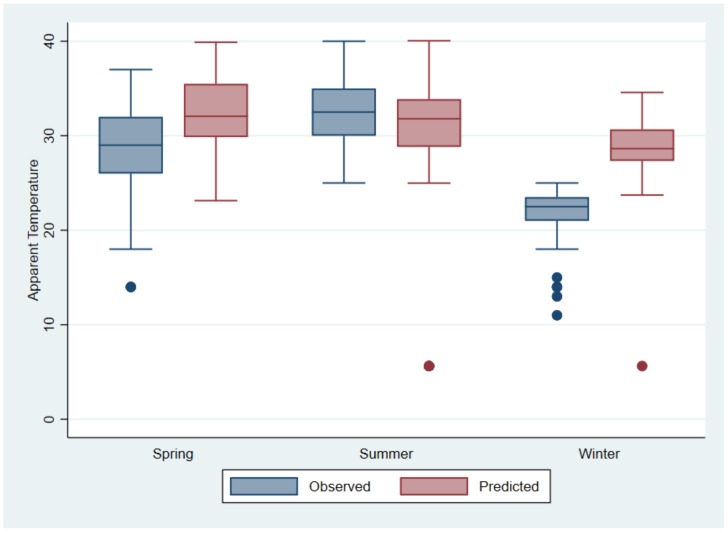
Boxplot of observed indoor AT during the study period compared to predicted AT obtained from linear prediction model using projected outdoor temperature.

Figure 7 compares indoor observed and predicted AT (obtained from linear regression) for each season. It is evident that indoor AT is predicted to increase significantly in spring (average increase of 3.9 °C) and winter (average increase of 7.1 °C) compared to summer (average increase of 2.0 °C). Therefore, people may be at increased risk of adverse health effects due to high AT in homes not only in summer when high temperatures are expected, but also in the previously cooler seasons of spring and winter.

Table 5 shows the number of days for the study period that fall within the four symptom bands using observed and predicted indoor AT. There are more days during which predicted future indoor AT falls within the caution and extreme caution bands compared to the number of days during which observed AT falls within those same bands. There are also fewer days in the future when household occupants are likely to be exposed to (predicted) indoor ATs that do not pose a health risk, therefore suggesting overall increased risk to high AT.

## 4. Discussion

A large percentage of households in the study area live below the upper bound of the poverty line, with many homes receiving financial assistance from the state in the form of grants for children and the elderly. Epidemiological studies on heat and health indicate that people residing in low-income districts with low socio-economic status are at highest risk of adverse health effects due to high temperatures [26]. A study conducted in Seoul, Korea, by Kim and Joh [59] to assess the effect of an economic factor in the association between mortality and high temperature, found an increase of 1.3–1.7 fold in the mortality rate of the low-income group compared to that of the general population. Similarly, Curriero et al [60] found that the percentage of poverty was the strongest predictor of mortality risk at temperatures higher than minimum mortality temperature in 11 cities across the Eastern United States.

The ability of households to alleviate heat stress depends on the assets they can afford, such as fans and air conditioners. Only 4.2% of homes sampled in the current study used air conditioners in hot weather and only 39.2% used fans. This shows that the majority of homes are likely exposed to high temperatures that could affect the health of occupants due to the lack of resources to adapt to heat. This situation will be exacerbated in the future based on projections of ambient temperature and indoor AT in the study area that show increases in the number of hot days when heat advisory warnings are likely to be issued by the national weather broadcasting service.

The most prevalent perception of indoor conditions during hot weather was that households felt hotter indoors than outdoors (48%). A study on the perceptions of thermal comfort in classrooms in South Africa found that the majority of students reported having at least one perceived heat-related health symptom when indoor temperatures were greater than or equal to 32 °C [61]. Diurnal patterns showed that indoor AT in the sampled homes often exceeded 32 °C in summer and spring, therefore it is possible that occupants could be experiencing perceived heat-related symptoms such as tiredness, low concentration, and breathing difficulties. A limitation of our study was that only one logger was placed in each household to obtain a representation of indoor temperature and AT conditions.

Similar to Smargiassi et al [62] and White-Newsome et al [63], we assessed the capability of outdoor conditions to predict indoor heat exposure in the form of AT. Regression results showed that ambient temperature can be used to predict indoor AT. This will be beneficial in estimating health effects of high temperatures in areas where there are currently no data on indoor conditions because data on ambient temperature is more readily available than indoor data.

Predicted outdoor temperature using monthly projections provided by the RCP climate model (8.5) showed that temperature in the study area located in Limpopo province will exceed the global mean annual surface air temperature (GMAT) increase of 1.5–2 °C. Our results show that for the period 2080–2099, ambient temperatures will increase by a mean of 4.6 °C. Previous studies using the same climate model have found that in Limpopo, temperatures will increase by 4–7 °C for the period 2080–2100 [18,64]. This increase will reach a regime never observed before in the recorded climate of this region. It is therefore evident that although GMAT is relevant for global scale policy recommendations, it is not necessarily a helpful metric in a South African setting for regional planning.

There were several hours of the day in summer during which calculated hourly AT rose to above 40.6 °C, which is the threshold at which the USNWS issues a heat advisory to warn people of the possibility of adverse health effects due to heat if precautions are not taken [51]. The high indoor AT observed during the study are cause for concern because a recent study using AT to assess heat exposure found a notable increase in daily mortality for each 1 °C increase in mean AT during the warm season [65]. Furthermore, warming in winter was projected to be greater than warming in summer and spring. This is similar to observations reported by the South African Weather Service [66].

Predicted indoor AT were grouped according to the number of days that will fall within the four symptom bands from the USNWS heat index. The number of days during which indoor AT will be categorized in the more severe symptom bands (caution and extreme caution) increases significantly. Conversely, the number of days during which average daily indoor AT will not pose a health risk decrease. This is supported by studies that project that there will be fewer cold days in the future [64]. Our results show that household occupants in the study area will be exposed to higher indoor AT that will have greater risk of adverse health impacts including heat cramps, heat exhaustion, and heat stroke.

The high measured and projected outdoor and indoor temperatures emphasize the need for national and provincial comprehensive heat response plans. These plans should include forecasting and monitoring to enable relevant authorities to warn planning agencies and communities timeously of imminent extreme temperature events [67]. They should also include education and awareness programs to inform communities of potential risk factors and high-risk population groups (i.e., being elderly or very young, having pre-existing chronic conditions, using certain medication, having restricted mobility), a description of symptoms of exposure to heat and recommended responses and treatments (e.g., avoid direct sunlight, wear light-weight and light colored clothing, drink water frequently, reduce or avoid strenuous activity outdoors if possible) [68]. Due to the high cost of purchasing and running air-conditioning units, the possibility of establishing emergency cooling shelters should be investigated. Studies have shown that spending only a few hours in an air-conditioned environment reduces heat-related illnesses and death [69].

## 5. Conclusions

Certain sectors of the population are more vulnerable to heat-related illnesses than others, particularly those with pre-existing health conditions, the elderly, and young children—as well as economically disadvantaged communities. This study aimed to consider the relationship between indoor and outdoor environments in rural residential settings in a current climate and warmer predicted future climate. Household occupants in the study area were exposed to high indoor AT, especially in summer and spring. The potential health risk of exposure to high indoor AT is likely to be exacerbated in future scenarios. The evidence of current and future adverse health effects will indicate priorities for planned adaptive strategies and will strengthen the case for pre-emptive policies. Such observations can be used to inform the development and implementation of policies and practices around heat and health, especially in rural areas of South Africa.

## Figures and Tables

**Table 1:** Apparent temperature ranges and associated possible heat health effects according to the USNWS NOAA Heat Index.

Level	Apparent Temperature Range	Warning	Possible Heat Health Effect
1	26.7–31.7 °C	Caution	Fatigue, discomfort possible
2	32.2–40 °C	Extreme caution	Sunstroke, heat cramps, heat exhaustion possible
3	40.6–53.9 °C	Danger	Sunstroke, heat cramps, heat exhaustion likely, heat stroke possible
4	54.5 °C or higher	Extreme danger	Sunstroke and heat stroke highly likely

**Table 2:** Responses to questionnaire: socio-demographic and house/household characteristics of sampled homes (*n* = 406).

Variables	*n*	%
Number of people in household		
<5	195	48.03
5–7	162	39.90
8–10	45	11.08
>10	4	0.99
Total	406	100.00
Number of children <5 years in household		
0	237	58.37
1	128	31.53
2	39	9.61
3	2	0.49
Total	406	100.00
Average monthly income of household—excluding grants and pensions		
No income	121	29.66
ZAR ≤ 1000	96	24.02
ZAR1001–ZAR5000	100	24.51
≥ZAR > 5001	17	4.17
Do not know	72	17.65
Total	406	100.00
* Number of people receiving the following grants		
Old age pension	142	
Disability	15	
Child	282	
Other	11	
When the weather is warm, how does the house feel indoors?		
Cooler than outside	107	26.35
Same as outside	104	25.62
Warmer than outside	195	48.03
Total	406	100.00
Do you use a fan during hot weather?		
Yes	159	39.16
No	247	60.84
Total	406	100.00
Do you use an air conditioner during hot weather?		
Yes	17	4.19
No	389	95.81
Total	406	100.00
Housing type		
Formal house	376	92.61
Informal dwelling	12	3.00
Traditional dwelling	16	3.90
Backyard dwelling (formal)	2	0.50
Total	406	100

* Response is total number people per household.

**Table 3:** Descriptive statistics on outdoor and indoor temperature, apparent temperature (AT), relative humidity (RH), and wind speed.

Season	Spring		Summer		Winter	
	Mean (SD)	Range	Mean (SD)	Range	Mean (SD)	Range
Outdoor Temperature (°C)	30.8 (5.3)	18.5–40.7	30.3 (4.8)	21.3–41.3	26.4 (3.1)	19.2–33.4
Outdoor AT (°C)	32.5 (8.5)	14.4–49.2	33.8 (8.4)	15.7–52.8	27.5 (5.3)	14.0–37.5
Outdoor RH (%)	78.4 (10.9)	54.0–95.0	90.9 (6.1)	70.0–96.0	85.3 (6.6)	66.0–96.0
Outdoor wind speed (m/s^−1^)	8.7 (2.9)	4.3–18.8	8.2 (3.0)	2.9–15.1	6.7 (2.4)	3.2–12.7
Indoor Temperature (°C)	27.2 (4.1)	16.0–34.9	28.5 (3.0)	22.8–35.6	22.1 (2.3)	13.5–25.8
Indoor AT (°C)	28.4 (5.1)	14.2–36.7	32.2 (3.4)	24.6–40.0	21.8 (2.8)	11.1–24.5
Indoor RH (%)	43.1 (9.1)	25.6–66.9	60.7 (8.8)	37.5–75.5	42.5 (8.4)	22.7–60.3

**Table 4:** Summary statistics from multiple regression using ambient data to find predictor(s) of indoor AT.

Variables		Coefficient	SE	*p* Value	95% CI
Dependent	Independent				
Indoor AT	Outdoor Temperature	0.55	0.17	0.001	0.23–0.88
	Outdoor AT	0.12	0.10	0.20	−0.07–0.33

SE = standard error, CI = confidence interval.

**Table 5:** Number of days within USNWS NOAA heat index symptom bands during current observed indoor AT conditions and future predicted indoor AT conditions.

US NWS Classification	Apparent Temperature Range	Observed AT (Number of Days)	Predicted AT (Number of Days)	Possible Heat Health Effect
No risk	<26.7 °C	97	23	-
Caution	26.7–31.7 °C	78	107	Fatigue, discomfort possible
Extreme caution	32.2–40.0 °C	61	106	Sunstroke, heat cramps, heat exhaustion possible
Danger	40.6–53.9 °C	0	0	Sunstroke, heat cramps, heat exhaustion likely, heat stroke possible
Extreme danger	54.5 °C or higher	0	0	Sunstroke and heat stroke highly likely
